# State of mental health, sleep status, and the interaction with health-related quality of life in HIV-infected Chinese patients during the COVID-19 pandemic

**DOI:** 10.1186/s12889-024-18929-5

**Published:** 2024-05-30

**Authors:** Juan Du, Jin Li, Han Liang, Fuxiang Wang, Yuanlong Lin, Bing Shao

**Affiliations:** 1 School of Pharmacy, Jilin medical university, Jilin, China; 2 Department of AIDS, Changchun Infectious Disease Hospital, Changchun, China; 3 Department of infectious diseases, Jilin Infectious Disease Hospital, Jilin, China; 4 The Third Department of Infection, Shenzhen Third People’s Hospital, Shenzhen, China; 5 School of Public Health, Jilin Medical University, Jilin, China

**Keywords:** AIDS, Mental health, Sleep status, Quality of life

## Abstract

**Objective:**

To describe how mental health and sleep status influence the health-related quality of life (HRQOL) of people living with HIV/AIDS (PLWHA) during the novel coronavirus disease 2019 (COVID-19) pandemic, and to apply targeted interventions to improve the HRQOL.

**Methods:**

A web-based online questionnaire survey was administered. Descriptive analysis was used to depict the mental health and sleep status. Correlation analysis and the structural equation model (SEM) method were used to analyze the influence of mental health and sleep status on HRQOL in PLWHA.

**Results:**

After excluding 24 unqualified questionnaires, a total of 490 participants in this survey were included in the statistical analysis. Of the participants, 66.1% and 55.1% reported mild or worse symptoms of depression and anxiety, respectively. Overall, 70.0% had varying degrees of sleep problems. Correlation analysis showed that anxiety had the strongest correlation with sleep disturbances and sleep quality (*R* = 0.588 and 0.551, respectively), while depression had the strongest correlation with the HRQOL psychological and physical domains (*R* = − 0.759 and − 0.682, respectively). SEM analysis showed that depression, sleep quality, and psychological domains had the greatest item load on mental health, sleep status, and HRQOL (093, 0.82, and 0.89, respectively). Mental health had a more significant influence than sleep status on HRQOL, as indicated by factor loading (− 0.75 and − 0.15, respectively).

**Conclusions:**

There were more severe mental health and sleep problems among PLWHA during the COVID-19 pandemic, thus, mental health intervention, especially to relieve depression symptoms, may be the most important approach to improve the HRQOL among PLWHA.

**Supplementary Information:**

The online version contains supplementary material available at 10.1186/s12889-024-18929-5.

## Introduction

By the end of 2020, the number of human immunodeficiency virus (HIV) infection survivors in China had reached 1.053 million, and the cumulative number of reported deaths was 351,000 [1]. Because acquired immune deficiency syndrome (AIDS) has become a controllable disease and the number of people living with HIV/AIDS (PLWHA) continues to increase, so more attention has been paid to the health-related quality of life (HRQOL) among PLWHA [2]. Indeed, it has been shown that PLWHA often have a lower HRQOL than healthy people [3]. Many factors have been reported to affect the HRQOL of PLWHA, such as age, educational status, social support, economic status, stigma, CD4 lymphocyte count, antiretroviral therapy, and body mass index [4–9]. Intervention measures target these factors will contribute to improve the HRQOL for PLWHA.

Since the end of 2019, the outbreak of novel coronavirus disease 2019 (COVID-19) has profoundly changed people’s lifestyles and behaviors [10], also significantly influenced the PLWHA, who are a special sub-population in society. The emergency prevention and control of COVID-19 requires a substantial amount of healthcare resources. It may be difficult for PLWHA patients to receive HIV-related treatment and regular medical care due to the restrictions imposed to control the COVID-19 epidemic, especially when PLWHA are infected with COVID-19. It has been previously reported that mental health problems are common in PLWHA [11]. During the COVID-19 global pandemic mental health problems increased among the global population, including the general population and populations with specific conditions [12, 13]. In this context, the mental health and sleep status of PLWHA warrant more attention. With the rapid development and growth of the Chinese economy and society, sleep disorders have become a general problem faced by an increasing number of people. According to the 2019 China Sleep Quality Survey Report, 83.81% of the respondents were frequently troubled by sleep problems [14]. PLWHA also face sleep problems due to their unique health issues [15]. The incidence of psychological and sleep problems in HIV-infected Chinese patients has been reported to be high; specifically, approximately three-fifths (60.3%) of PLWHA reported poor sleep quality, 50.0% and 36.3% exhibited depression and anxiety symptoms, respectively [16, 17]. We speculate that during the COVID-19 pandemic, with concerns about the epidemic and restrictive measures implemented to control the COVID-19 epidemic, many people including PLWHA were at risk of unemployment and loss of financial resources or were affected by the prolonged lockdown measures. Thus, the psychological and sleep problems would be much more severe than before. The possible association between the psychological effect and sleep disturbances have attracted attention [18, 19]. The HRQOL of PLWHA may be inevitably affected by these factors and are worthy of consideration during the COVID-19 pandemic.

The purpose of this study was to describe the mental health and sleep status, and HRQOL among PLWHA in China during the COVID-19 pandemic, analyze how mental health and sleep status influence HRQOL among PLWHA, and propose targeted interventions to improve HRQOL, especially during the infectious disease pandemics such as COVID-19.

## Methods

### Participants and study design

A cross-sectional study was designed, and a web-based questionnaire survey was administered to PLWHA in China. The questionnaire was compiled through the “Survey Star” online platform, then distributed to the patients’ WeChat groups via their clinicians from the cities of Changchun, Beijing, and Shenzhen. The content of the questionnaire included the participants’ basic demographic characteristics, HRQOL, and mental health status, such as anxiety, depression, and sleep status. The demographic characteristics included date of birth, gender, household registration, city of medical treatment, marital status, education level, infection route, occupation, monthly income, treatment status, and recent CD4 + T lymphocyte count. The HRQOL among PLWHA was based on the brief version of the World Health Organization quality of life questionnaire for HIV (WHOQOL-HIV-BRIEF), which consists of six domains (physical, independence, relationship, spirituality, psychological, and environment). The questionnaire scale of HRQOL has been widely used before [20, 21].

The Zung self-rating anxiety scale (SAS) and Zung self-rating depression scale (SDS) were used to determine the anxiety and depression status among PLWHA, respectively. SAS and SDS consist of 20 items each. The scores of the 20 items are added to yield a rough total score, then multiplied by 1.25 to obtain the integer for the standard score. Higher scores correspond to more severe symptoms. SAS scores are categorized as follows: < 50, normal; 50–59, mild; 60–69, moderate; and > 69, severe anxiety. SDS scores are categorized as follows according to the standard of Chinese norms [22–24]: < 53, normal; 53–62, mild; 63–72, moderate; and > 72 severe depression. The usage of SAS and SDS has been described detailedly in study [25], and widely used and confirmed to have adequate reliability and validity in previous studies [5, 22].

Sleep status was investigated and assessed using the Pittsburgh sleep quality index (PSQI) scale, which was used to assess the sleep status of the participants in the last month. This self-report questionnaire has 19 items, of which 18 items participate in scoring and forming seven components, including sleep duration, sleep efficiency, sleep latency, sleep disturbances, sleep quality, use of sleeping medications, and daytime dysfunction. The cumulative score of each domain is the total PSQI score, and the total score ranges from 0 to 2 l, with higher scores corresponding to worse sleep status. Based on the manual of the index, a score *≥* 5 is defined as poor quality sleep, scores from 0 to 5 are defined as “very good sleep,” scores from 6 to 10 are defined as “not too bad” sleep status, scores from 11 to 15 are defined as “generally poor” sleep status, and scores from 16 to 21 are defined as “poor sleep” [26, 27]. The reliability and validity of the PSQI used in Chinese PLWHA has been confirmed previously [28].

The respondents had no special restrictions. The respondents had to be diagnosed with an HIV infection or AIDS, voluntarily participated in the study, and had the physical and mental ability to complete the questionnaire using a web-based procedure program. The purpose and significance of the survey was explained to the potential respondents before the questionnaire was distributed, and the respondents were informed that the survey was based on the principles of voluntary participation, confidentiality, and respect. The respondents volunteered to participate in the study and complete the questionnaires according to their own actual situation. Finally, the names of the respondents were not recorded on the questionnaires. Based on these principles, all participants were not required to sign an informed consent document. The survey carried out from May–September 2021. A total of 514 PLWHA from 28 provinces or autonomous regions and municipalities participated in the survey. After excluding unqualified questionnaires, such as censored data, a total of 490 respondent questionnaires were included and underwent subsequent statistical analysis, and all data were de-identified and analyzed anonymously. The study has been approved by the Medical Ethics Committee of Jilin Medical University.

### Data analysis

Descriptive analysis was used to reveal the basic characteristics and state of mental health and sleep status among PLWHA. Pearson correlation analysis was used to analyze the correlation among mental health status, each PSQI domain, and HRQOL using SPSS 26.0 software (IBM Corp., Armonk, NY, USA ). A structural equation model (SEM) was built to examine the relationship among mental health, PSQI, and HRQOL, after which the factor loading of each domain on these three dimensions, and the path coefficient among mental health, PSQI, and HRQOL were calculated. A SEM diagram was drawn with SPSS AMOS 23.0 statistical software (IBM Corp.).

## Results

### Characteristics of the participants

Most participants (38.8%) were 30–39 years of age and 94.7% of the participants were male. The main route of infection was attributed to homosexual activity (80.6%), followed by heterosexual activity (9.4%). Urban household registration accounted for 61.8% of the participants, most of whom were single (59.4%). The income per month was < 5000 RBM in the majority of participants (61.5%). Most of the participants were civil servants or enterprise personnel (25.7%; Table 1).

**Table 1:** Basic demographic characteristics of participants

Variables	Number	
	(*N* = 490)	%
Age		
< 30	127	25.90
30–39	190	38.80
40–49	113	23.10
≥ 50	60	12.20
Genders		
Male	464	94.70
Female	26	5.30
Routes of infection		
Homosexual	395	80.60
Heterosexual	46	9.40
Intravenous drug	3	0.60
Blood/Blood Products	12	2.40
Mother-to-child transmission	0	0.00
Others	34	6.90
Census register		
City	303	61.80
Country	187	38.20
Marital status		
Single	291	59.40
Married	128	26.10
Divorced	67	13.70
Widowed	4	0.80
Educational level		
Below junior high school	65	13.30
High School/technical secondary School	128	26.10
College or above	264	53.90
Graduate and above	33	6.70
Incomes		
< 3000	136	27.80
3000–5000	165	33.70
5000–10,000	131	26.70
> 10,000	58	11.80
Profession		
Farmer	23	4.70
Student	28	5.70
Civil servant, enterprises personnel	126	25.70
Self-employed	55	11.20
Others	258	52.70

Of the participants, 66.1% and 55.1% had mild or worse depression and anxiety symptoms, respectively, of whom 11.6% and 12.7% had severe depression and anxiety symptoms, respectively. Only 30.0% of the participants had good sleep status and 70.0% had varying degrees of sleep problems, of whom 23.7% had general or very poor sleep status. The mean and SD for each grade of depression, anxiety and sleep status were also presented, which showed that the higher the score, the worse the mental status and sleep quality. (Table 2).

**Table 2:** State of mental health and sleep status of participants

	Number	%	Mean	SD
Depression				
Not	166	33.9	42.2	0.6
Mild	143	29.2	58.5	0.2
Moderate	124	25.3	67.0	0.3
Severe	57	11.6	79.4	0.6
Anxiety				
Not	220	44.9	39.6	0.5
Mild	125	25.5	54.1	0.3
Moderate	83	16.9	63.4	0.3
Severe	62	12.7	78.1	0.9
PSQI				
Good	147	30.0	3.8	0.1
Not too bad	227	46.3	7.9	0.1
General	97	19.8	12.9	0.1
Very poor	19	3.9	17.9	0.3

### Correlation of each domain among mental health, PSQI, and HRQOL

Pearson correlation analysis showed that anxiety and depression were positively correlated with each domain of the PSQI. The correlation coefficient between anxiety and sleep disturbances and sleep quality were maximal (0.588 and 0.551, respectively). Anxiety and depression had the strongest correlation with the psychological and physical domains of the HRQOL, the correlation coefficients were maximal between depression, and psychological and physical domains (− 0.759 and − 0.682, respectively; Table 3).

**Table 3:** Correlation analysis of each domain of mental health, sleep status and HRQOL

		Physical	Independence	Relationship	Spirituality	Psychological	Environment	Depression	Anxiety	Sleep duration	Sleep efficiency	Sleep latency	Sleep disturbances	Sleep quality	Use of sleeping medication	Daytime dysfunction
Physical	Pearson	1.000														
	P-value															
Independence	Pearson	**0.693**	1.000													
	P-value	< 0.001														
Relationship	Pearson	0.592	0.577	1.000												
	P-value	< 0.001	< 0.001													
Spirituality	Pearson	0.584	0.444	0.540	1.000											
	P-value	< 0.001	< 0.001	< 0.001												
Psychological	Pearson	0.704	0.684	0.695	0.589	1.000										
	P-value	< 0.001	< 0.001	< 0.001	< 0.001											
Environment	Pearson	0.673	0.657	0.732	0.520	0.756	1.000									
	P-value	< 0.001	< 0.001	< 0.001	< 0.001	< 0.001										
Depression	Pearson	**-0.682**	-0.615	-0.580	-0.549	**-0.759**	-0.657	1.000								
	P-value	< 0.001	< 0.001	< 0.001	< 0.001	< 0.001	< 0.001									
Anxiety	Pearson	**-0.666**	-0.562	-0.517	-0.514	**-0.671**	-0.580	**0.817**	1.000							
	P-value	< 0.001	< 0.001	< 0.001	< 0.001	< 0.001	< 0.001	< 0.001								
Sleep duration	Pearson	**-0.278**	-0.193	-0.221	-0.079	-0.198	-0.204	0.208	0.180	1.000						
	P-value	< 0.001	< 0.001	< 0.001	0.082	< 0.001	< 0.001	< 0.001	< 0.001							
Sleep efficiency	Pearson	**-0.237**	-0.191	-0.181	-0.079	-0.176	-0.162	0.219	0.234	0.591	1.000					
	P-value	< 0.001	< 0.001	< 0.001	0.08	< 0.001	< 0.001	< 0.001	< 0.001	< 0.001						
Sleep latency	Pearson	**-0.416**	-0.352	-0.298	-0.298	-0.386	-0.359	0.427	0.464	0.278	0.360	1.000				
	P-value	< 0.001	< 0.001	< 0.001	< 0.001	< 0.001	< 0.001	< 0.001	< 0.001	< 0.001	< 0.001					
Sleep disturbances	Pearson	**-0.503**	-0.454	-0.365	-0.366	**-0.446**	-0.396	**0.516**	**0.588**	0.249	0.273	0.479	1.000			
	P-value	< 0.001	< 0.001	< 0.001	< 0.001	< 0.001	< 0.001	< 0.001	< 0.001	< 0.001	< 0.001	< 0.001				
Sleep quality	Pearson	**-0.614**	-0.442	-0.399	-0.361	**-0.477**	-0.419	**0.502**	**0.551**	0.397	0.403	0.575	0.541	1.000		
	P-value	< 0.001	< 0.001	< 0.001	< 0.001	< 0.001	< 0.001	< 0.001	< 0.001	< 0.001	< 0.001	< 0.001	< 0.001			
Use of sleeping medication	Pearson	**-0.204**	-0.162	-0.135	-0.114	-0.154	-0.141	0.166	0.256	0.193	0.260	0.314	0.248	0.331	1.000	
	P-value	< 0.001	< 0.001	0.003	0.012	0.001	0.002	< 0.001	< 0.001	< 0.001	< 0.001	< 0.001	< 0.001	< 0.001		
Daytime dysfunction	Pearson	**-0.536**	-0.43	-0.394	-0.346	**-0.508**	-0.433	0.463	0.522	0.195	0.207	0.387	0.435	0.504	0.244	1.000
	P-value	< 0.001	< 0.001	< 0.001	< 0.001	< 0.001	< 0.001	< 0.001	< 0.001	< 0.001	< 0.001	< 0.001	< 0.001	< 0.001	< 0.001	

Each domain of the PSQI was negatively correlated with the HRQOL domains, but showed the strongest correlation with the HRQOL physical domain. The correlation between sleep quality and physical domain was the strongest (− 0.614), followed by daytime dysfunction (− 0.536), and sleep disturbances (− 0.503). Sleep quality, daytime dysfunction, and sleep disturbances also had a strong negative correlation with the HRQOL psychological domain; the correlation coefficients were − 0.477, − 0.508, and − 0.446, respectively. The correlation coefficient between the PSQI and HRQOL total score was − 0.557.(Table 3).

#### SEM analysis among mental health, PSQI, and HRQOL

**Fig. 1:**
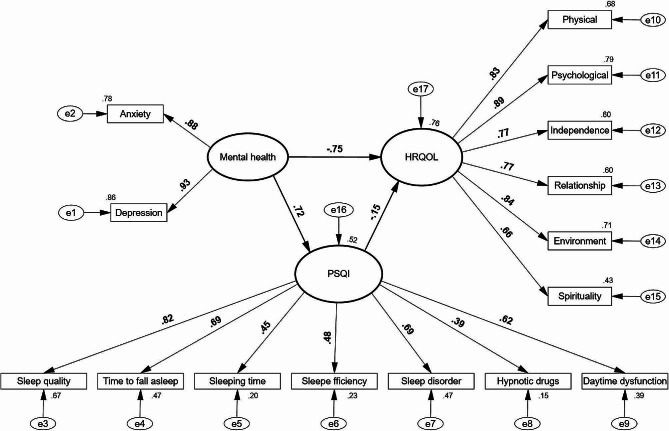
The structural equation model for the hypothesis that mental health domains and sleep status (PSQI) influence the health-related quality of life in people living with HIV/AIDS.

The SEM analysis results were as follows, which indicated that the model fit the data well: χ^2^ = 483.013; *p* < 0.001; root mean square error of approximation (RMSEA), 0.096; and goodness of fit (GFI), 0.883. The SEM results also showed that within the mental health dimension, depression had the largest factor loading (0.93). Sleep quality had the largest factor loading (0.82) within the PSQI. The HRQOL psychological domain had the largest factor loading (0.89), followed by the physical and environmental domains (0.83 and 0.84, respectively). The path coefficients of mental health on HRQOL and PSQI were − 0.75 and 0.72, respectively, while the path coefficient of sleep status on HRQOL was only − 0.15(Fig. 1).

## Discussion

With the COVID-19 global pandemic, mental health has become a common issue for the general public. People in different regions or countries have been reported to experience severe mental health and sleep problems during the COVID-19 pandemic [29, 30]. Although mental health and sleep status have been reported to impact the quality of life among PLWHA [31, 32], with the implementation of strict preventive policies during the COVID-19 epidemic in China, PLWHA may not only experience the mental stress from HIV infection and treatment, but also face stress from life and work, which might cause severe mental health or sleep problems for PLWHA than usual. Our results showed a higher incidence of anxiety and depression than previous studies conducted involving Chinese PLWHA prior to the COVID-19 outbreak. More severe mental health and sleep problems among PLWHA existed during COVID-19 epidemic based on the findings in current study [28, 33, 34]. The same results were also found in other regions outside China [35, 36]. More severe psychological and sleep problems during COVID-19 pandemic may result in more adverse effects on the HRQOL for PLWHA.

Correlation analysis showed that anxiety and depression had a strong positive correlation with sleep disturbances and sleep quality. Mental health status is usually considered to be a cause, rather than a consequence of sleep disturbance among HIV-infected patients [37, 38]. Anxiety had a closer correlation with sleep disturbances and sleep quality than depression among PLWHA. Anxiety and depression also had a significant negative correlation with the psychological and physical domains of HRQOL, but depression was more significantly associated with psychological health. In brief, anxiety may lead to more adverse effects on sleep status, while depression may more affect the psychological health among PLWHA.

The present study showed that all domains of the PSQI exhibited the strongest negative correlativity with the HRQOL physical domain. The most notable correlation was between sleep quality and the physical domain (coefficient, − 0.614). Moreover, daytime dysfunction, sleep disturbances and sleep quality of the PSQI also had a significant correlation with the HRQOL psychological domain. Studies revealed that improved sleep quality promotes improvement in the HRQOL among patients with HIV, and better sleep helps reduce anxious personality and depressive symptoms [39, 40]. Our study revealed that sleep quality, daytime dysfunction, and sleep disturbances can exert a greater effect on physical and psychological health among PLWHA. Intervention for sleep status is more effective in improving HRQOL, especially for taking measures to improve sleep quality, daytime dysfunction, and sleep disturbances not only benefit physical health, but also promote psychological health among PLWHA. Although some studies have identified influencing factors, such as social support and socioeconomic status on the quality of life among PLWHA [5, 41], intervention targeting social support and economic status may not be an effective measure that can achieve a short-term effect. Intervention strategies to improve mental health and sleep are feasible methods to evaluate the intervening effect in anytime because implementing mental health and sleep status interventions are likely to be quick and facilitated to improve the quality of life among PLWHA.

The SEM showed that the HRQOL psychological, environmental, and physical domains exhibited the largest factor loadings, indicating psychological, environmental, and physical health status among PLWHA had the most significant influence on the overall HRQOL. The significant feature of the environmental domain influence on HRQOL suggests that improving the environmental health among PLWHA may also be an important measure to improve HRQOL. This viewpoint is in agreement with a previously published finding [4] that emphasized the important role of the environmental domain on influencing the quality of life among PLWHA. In addition, the results of the SEM analysis revealed that the path coefficient of the HRQOL mental health domain **(**− 0.75**)** was far greater than the HRQOL sleep status domain (− 0.15). When compared with sleep status, intervention for mental health will have a greater effect on improving the quality of life among PLWHA. Moreover, considering that the depression and psychological domains had the largest loading factors in mental health and the HRQOL, and a strong positive correlation existed between depression and the psychological domain according to the results of correlation analysis, interventions for depression among PLWHA should be the most effective manner for improving HRQOL. Interventions for depression will have a direct role in improving the HRQOL psychological health domain, and can be regarded as the most effective way to improve the quality of life among PLWHA.

The present study was conducted using a web-based online survey. This online-based survey of PLWHA is very popular [30, 42, 43], and during the COVID-19 pandemic, this survey method was seemingly most appropriate and useful, because it was convenient for PLWHA to respond and reply using a web-based method under the COVID-19 strict control measures. Moreover, this method can also protect the privacy issues for this special population. Overall, it was possible to obtain accurate information compared to an off-line face-to-face mode; however, the obtained samples may have been subject to selection bias. During the survey, although the clinicians informed their patients to cooperate as much as possible to complete the questionnaires, some candidates may still not take part according to the principle of voluntary participation, leading to the study were more made up of voluntary samples. In conclusion, the results of this study showed the significance of mental health on sleep status and quality of life among PLWHA. Interventions for depression among PLWHA are the most effective manner to improve HRQOL and promote psychological health. Given the high incidence of depressive symptoms among Chinese PLWHA [34, 44], treatment, if supplemented with mental health interventions, such as controlling or alleviating depression symptoms, will achieve a better treatment effect. Physicians should not only focus on the results of antiviral treatment of patients, but also pay more attention to their mental health status and take appropriate intervention measures, which will improve the quality of life among PLWHA, especially during the infectious disease pandemic such as COVID-19.

### Electronic supplementary material

Below is the link to the electronic supplementary material.

Supplementary Material 1

## Data Availability

The datasets used and/or analyzed during the current study are available from the corresponding author on reasonable request.
