# Supplementary material for: State of mental health, sleep status, and the interaction with health-related quality of life in HIV-infected Chinese patients during the COVID-19 pandemic
Source: BMC Public Health. 2024 May 30;24:1445. doi: 10.1186/s12889-024-18929-5 (PMC11137900; doi:10.1186/s12889-024-18929-5)
Supplement: Supplementary file 1 — Supplementary Material 1 [file 12889_2024_18929_MOESM1_ESM.docx]

Questionnaire

Part I. The basic information of participators

Please complete the answers or make the choices that best for you according to your actual situations.

1.Your name :____________(can be replaced by letters and other symbols that you can recognize)

2.Your date of birth: _________Year_________Month

3.Your gender： ➀ Male ➁Female

4.Your nationality：____________

5.Do you have religious beliefs： ➀Yes ➁No

6.What is your census register? ➀Urban ➁Rural

7.Your marital status:

➀Unmarried ➁Married ➂Divorced ➃Widowed

8. Where are you seeking medical treatment now____________

9.Your educational level :

➀Below junior high school ➁High School/technical secondary School

➂College or above ➃Graduate and above

10.Your average monthly income (RMB)

➀ Less than 3000 ➁3000-5000 ➂5000-10000 ➃ More than 10000

11.Your current occupation:

➀Farmer ➁Student ➂Civil servant, enterprises personnel

➃Self-employed ➄Others

12.When were you first diagnosed with HIV? __________Year________Month

13.Routes of HIV-infection

➀Homosexual ➁Heterosexual ➂Intravenous drug

➃Blood or Blood Products ➄Mother-to-child transmission ➅Others

14.Do people around you (relatives, friends or colleagues) know that you are HIV-positive? ➀Yes ➁No

15. Are you currently receiving antiretroviral treatment for HIV?

➀Yes ➁No (skip to Question 18)

16. Have you taken the medicine according to the dosage and time prescribed by your doctor in the last one week? ➀Yes ➁No

17.How long have you been taking the medication so far?

➀1-3 months ➁3-6 months ➂6-12 months ➃More than 12 months

18.How much of your last CD4 count?

➀Less than 100 ➁100-350 ➂More than 350

**Part Ⅱ. WHOQOL-HIV BREF**

**This assessment asks how you feel about your quality of life, health, or other areas of your life. We ask that you think about your life in the last two weeks. Please answer all the questions. If you are unsure about which response to give to a question, please choose the one that appears most appropriate.**

**Please read each question, assess your feelings, and place a check mark on the number for each question that gives the best answer for you.**

1. How would you rate your quality of life?

➀Very poor ➁Poor ➂Neither poor nor good ➃Good ➄Very good

1. How satisfied are you with your health?

➀Very dissatisfied ➁Dissatisfied ➂Neither satisfied nor dissatisfied

➃Satisfied ➄Very satisfied

1. To what extent do you feel that physical pain prevents you from doing what you need to do?

➀Not at all ➁A little ➂A moderate amount

➃Very much ➄An extreme amount

1. How much are you bothered by any physical problems related to your HIV infection?

➀Not at all ➁A little ➂A moderate amount

➃Very much ➄An extreme amount

1. How much do you need any medical treatment to function in your daily life?

➀Not at all ➁A little ➂A moderate amount

➃Very much ➄An extreme amount

1. How much do you enjoy life?

➀Not at all ➁A little ➂A moderate amount

➃Very much ➄An extreme amount

1. To what extent do you feel your life to be meaningful?

➀Not at all ➁A little ➂A moderate amount

➃Very much ➄An extreme amount

1. To what extent are you bothered by people blaming you for your HIV status

➀Not at all ➁A little ➂A moderate amount

➃Very much ➄An extreme amount

1. How much do you fear the future?

➀Not at all ➁A little ➂A moderate amount

➃Very much ➄An extreme amount

1. How much do you worry about death?

➀Not at all ➁A little ➂A moderate amount

➃Very much ➄An extreme amount

1. How well are you able to concentrate?

➀Not at all ➁A little ➂A moderate amount ➃Very much ➄Extremely

1. How safe do you feel in your daily life?

➀Not at all ➁A little ➂A moderate amount ➃Very much ➄Extremely

1. How healthy is your physical environment?

➀Not at all ➁A little ➂A moderate amount ➃Very much ➄Extremely

**The following questions ask about how completely you experience or were able to do certain things in the last two weeks.**

1. Do you have enough energy for everyday life?

➀Not at all ➁A little ➂Moderately ➃Mostly ➄Completely

1. Are you able to accept your bodily appearance?

➀Not at all ➁A little ➂Moderately ➃Mostly ➄Completely

1. Have you enough money to meet your needs?

➀Not at all ➁A little ➂Moderately ➃Mostly ➄Completely

1. To what extent do you feel accepted by the people you know?

➀Not at all ➁A little ➂Moderately ➃Mostly ➄Completely

1. How available to you is the information that you need in your day-to-day life?

➀Not at all ➁A little ➂Moderately ➃Mostly ➄Completely

1. To what extent do you have the opportunity for leisure activities?

➀Not at all ➁A little ➂Moderately ➃Mostly ➄Completely

1. How well are you able to get around?

➀Very poor ➁Poor ➂Neither poor nor good ➃Good ➄Very good

**The following questions ask you how good or satisfied you have felt about various aspects of your life over the last two weeks.**

1. How satisfied are you with your sleep?

➀Very dissatisfied ➁Dissatisfied ➂Neither satisfied nor dissatisfied

➃Satisfied ➄Very satisfied

22. How satisfied are you with your ability to perform your daily living activities?

➀Very dissatisfied ➁Dissatisfied ➂Neither satisfied nor dissatisfied

➃Satisfied ➄Very satisfied

1. How satisfied are you with your capacity for work?

➀Very dissatisfied ➁Dissatisfied ➂Neither satisfied nor dissatisfied

➃Satisfied ➄Very satisfied

1. How satisfied are you with yourself?

➀Very dissatisfied ➁Dissatisfied ➂Neither satisfied nor dissatisfied

➃Satisfied ➄Very satisfied

1. How satisfied are you with your personal relationships?

➀Very dissatisfied ➁Dissatisfied ➂Neither satisfied nor dissatisfied

➃Satisfied ➄Very satisfied

1. How satisfied are you with your sex life?

➀Very dissatisfied ➁Dissatisfied ➂Neither satisfied nor dissatisfied

➃Satisfied ➄Very satisfied

27.How satisfied are you with the support you get from your friends?

➀Very dissatisfied ➁Dissatisfied ➂Neither satisfied nor dissatisfied

➃Satisfied ➄Very satisfied

28.How satisfied are you with the conditions of your living place?

➀Very dissatisfied ➁Dissatisfied ➂Neither satisfied nor dissatisfied

➃Satisfied ➄Very satisfied

29. How satisfied are you with your access to health services?

➀Very dissatisfied ➁Dissatisfied ➂Neither satisfied nor dissatisfied

➃Satisfied ➄Very satisfied

30.How satisfied are you with your transport?

➀Very dissatisfied ➁Dissatisfied ➂Neither satisfied nor dissatisfied

➃Satisfied ➄Very satisfied

**The following question refers to how often you have felt or experienced certain things in the last two weeks**

31.How often do you have negative feelings such as blue mood, despair, anxiety,depression? ➀Never ➁Seldom ➂Quite often ➃Very often ➄Always

**Part Ⅲ. Zung Self-Rating Depression Scale (SDS)**

**For each item below, please place a check mark in the column which best describes how often you felt or behaved this way during the past several days**

**A.** A little of the time (In the past week, there been no more than one day of such situation)

**B.** Some of the time(In the past week, there been 1-2 days of such situation)

**C.** Quite a lot of time (In the past week, there been 3-4 days of such situation)

**D.** Most of the time(In the past week, there been 5-6 days of such situation)

1. I feel down-hearted and blue. A B C D
2. Morning is when I feel the best A B C D
3. I have crying spells or feel like it. A B C D
4. I have trouble sleeping at night. A B C D
5. I eat as much as I used to. A B C D

6. I still enjoy sex. A B C D

7. I notice that I am losing weight. A B C D

8. I have trouble with constipation. A B C D

9. My heart beats faster than usual. A B C D

10. I get tired for no reason. A B C D

11. My mind is as clear as it used to be. A B C D

12. I find it easy to do the things I used to. A B C D

13. I am restless and can't keep still. A B C D

14. I feel hopeful about the future. A B C D

15. I am more irritable than usual. A B C D

16. I find it easy to make decisions. A B C D

17. I feel that I am useful and needed. A B C D

18. My life is pretty full. A B C D

19. I feel that others would be better off if I were dead. A B C D

20. I still enjoy the things I used to do. A B C D

**Part Ⅳ. Zung Self-Rating Anxiety Scale (SAS)**

**For each item below, please place a check mark in the column which best describes how often you felt or behaved this way during the past several days.**

**A.** A little of the time (In the past week, there been no more than one day of such situation)

**B.** Some of the time(In the past week, there been 1-2 days of such situation)

**C.** Quite a lot of time (In the past week, there been 3-4 days of such situation)

**D.** Most of the time(In the past week, there been 5-6 days of such situation)

1. I feel more nervous and anxious than usual. A B C D

2. I feel afraid for no reason at all. A B C D

3. 1 get upset easily or fed panicky. A B C D

4. 1 feel like Im falling apart and going to pieces. A B C D

5. I feel that everthing is all right and nothing bad will happen. A B C D

6. arms and legs shake and tremble. A B C D

7. I am bothered by headaches neck and back pain. A B C D

8. I feel weak and get tired easily. A B C D

9. I feel calm and can sit still easily. A B C D

10. I can feel my heart beating fast. A B C D

11. 1 am bothered by dizzy spells A B C D

12. I have fainting spells or feel like it A B C D

13. I can breathe in atxl out easily. A B C D

14. I get feelings of numbness and tingling in my fingers & toes. A B C D

15. I am bothered by stomach aches or indigestion. A B C D

16. I have to empty my bladder often. A B C D

17. My hands are usually dry and warm. A B C D

18. My face gets hot and blushes. A B C D

19. I fall asleep easily and get a good night's rest. A B C D

20. I have nightmares. A B C D

**PartⅤ. Pittsburgh Sleep Quality Index (PSQI)**

**The following questions relate to your usual sleep habits during the past month only. Your answers should indicate the most accurate reply for the majority of days and nights in the past month. Please answer all questions.**

1.During the past month, what time have you usually gone to bed at night?

BED TIME：_________

2. During the past month, how long (in minutes) has it usually taken you to fall asleep each night?

NUMBER OF MINUTES：_________

3. During the past month, what time have you usually gotten up in the morning?

GETTING UP TIME:_____________

4. During the past month, how many hours of actual sleep did you get at night? (This may be different than the number of hours you spent in bed.)

HOURS OF SLEEP PER NIGHT:____________

**For each of the remaining questions, check the one best response. Please answer all questions.**

5. How often during the past month have you had trouble sleeping because of this?

a.Cannot get to steep within 30 minutes

➀Not during the past month ➁Less than once a week

➂Once or twice a week ➃Three or more times a week

b.Wake up in the middle of the night or early morning

➀Not during the past month ➁Less than once a week

➂Once or twice a week ➃Three or more times a week

c.Have to get up to use the bathroom

➀Not during the past month ➁Less than once a week

➂Once or twice a week ➃Three or more times a week

d. Cannot breathe comfortably

➀Not during the past month ➁Less than once a week

➂Once or twice a week ➃Three or more times a week

e. Cough or snore loudly

➀Not during the past month ➁Less than once a week

➂Once or twice a week ➃Three or more times a week

f.Feel too cold

➀Not during the past month ➁Less than once a week

➂Once or twice a week ➃Three or more times a week

g. Feel too hot

➀Not during the past month ➁Less than once a week

➂Once or twice a week ➃Three or more times a week

h. Had bad dreams

➀Not during the past month ➁Less than once a week

➂Once or twice a week ➃Three or more times a week

i.Have pain

➀Not during the past month ➁Less than once a week

➂Once or twice a week ➃Three or more times a week

j. Other reason(s), if have, please describe ________________________

➀Not during the past month ➁Less than once a week

➂Once or twice a week ➃Three or more times a week

6. During the past month, how would you rate your sleep quality overall?

➀Very good ➁Fairly good ➂Fairly bad ➃Very bad

7. During the past month, how often have you taken medicine to help you sleep (prescribed or "over the counter")?

➀Not during the past month ➁Less than once a week

➂Once or twice a week ➃Three or more times a week

8. During the past month, how often have you had trouble staying awake while driving, eating meals, or engaging in social activity?

➀Not during the past month ➁Less than once a week

➂Once or twice a week ➃Three or more times a week

9. During the past month, how much of a problem has it been for you to keep up enough enthusiasm to get things done?

➀No problem at all ➁Only a very slight problem

➂Somewhat of a problem ➃A very big problem
